# An urgent call to address the nutritional status of women and children in Nepal during COVID-19 crises

**DOI:** 10.1186/s12939-020-01210-7

**Published:** 2020-06-05

**Authors:** Bindu Panthi, Pratik Khanal, Minakshi Dahal, Sajana Maharjan, Sushil Nepal

**Affiliations:** 1 Nobel College, Pokhara University, Kathmandu, Nepal; 2 Institute of Medicine, Tribhuvan University, Kathmandu, Nepal; 3 Center for Research on Environment, Health and Population Activities (CREHPA), Kathmandu, Nepal; 4 One Heart Worldwide, Kathmandu, Nepal

## Abstract

Due to the ongoing nationwide lockdown in Nepal, women and children face a greater risk of malnutrition and eventually leading to mortality and morbidity. To harness the progress made so far in improving the nutritional status of women and children, a focus on nutrition should be a part of the COVID-19 response plan.

## Background

As the threat of COVID-19 pandemic is expanding, numerous nations have confined development or actualized lockdowns to give their public health strategies more opportunity to focus on prevention and management of COVID-19 infections. The Government of Nepal has imposed nationwide lockdown from March 24 until June 14 in 2020 and might extend beyond [1]. Despite nationwide lockdown and expansion of testing facility, cases have upsurge from 2 on March 24 to 245 on May 13 [2]. While the lockdown can be considered as a significant move to battle the spread of the virus in the country considering its health system capacities, the effect of the prolonged lockdown will affect the lives of the population. Importantly, the effect on powerless portions of society can be cataclysmic, especially women and children as they are one of the most vulnerable groups during emergencies and disasters.

## Main text

The impact of the COVID-19 on nutrition outcomes has not been acknowledged at this point in Nepal but studies have shown that large scale emergencies have increased morbidity and often mortality in infants and young children [3–5]. These adverse outcomes are usually the result of low immunity, gastrointestinal or respiratory tract infections, and associated with malnutrition or dehydration [6]. Malnutrition and its human and economic cost are enormously falling hardest on poor women and children and lasting to generations. According to the Nepal’s demographic health survey 2016, wasting rate among under 5-year children in Nepal is 9.7% [7], and as per Nepal Micronutrient survey 2016, the prevalence of reported night-blindness among women aged 15–49 years during last pregnancy is 8.5%, and the prevalence of Vitamin A Deficiency among children aged 6–59 months is 4% [8]. Similarly, 53% of children under five and 69% of children aged 6–23 months and 41% of women of reproductive age group suffer from anemia [7] which is a public health concern according to the WHO [9].

Communities and vulnerable groups like women and children dealing with malnutrition are doubly susceptible to compromised health due to COVID-19 pandemic. That is because, malnutrition can weaken the immune system and they may be more vulnerable to acquiring COVID-19. In addition, the lockdown has resulted in a decrease in household incomes leading to less availability and reduced access to food, and restriction in receiving essential health care services. Nutrition services like vitamin A and deworming campaign, supplementation of micronutrient powders, treatment of malnourished children through the outpatient therapeutic center, and nutrition rehabilitation homes have also been affected as a result of the priority shift of health sector towards COVID-19. Alternatively, the pandemic could force people to take on behaviors like consumption of unhealthy food, inadequate intake of nutritious food, and poor hygiene and sanitation practice. Insufficient breastfeeding practices, due to fear and anxiety of transmission of COVID 19 from breastfeeding mothers, result in decreased feeding and caring practices for children. The predictable outcomes of such situations might decrease the immune function, increase the susceptibility to severe diseases, and lead to a high incidence of wasting and stunting among children.

Food insecurity and hunger is one of the top issues of malnutrition in Nepal. COVID-19 could further induce malnutrition due to people losing their jobs mainly informal sector and those in foreign employment. Similarly, it might also affect the agricultural production due to shortage of seeds, fertilizers and manpower, further plunging the population into poverty and malnutrition. According to Global Hunger Index 2019, Nepal experiences a serious threat of hunger, with a score of 20.8 standing at rank of 73 among 117 countries [10], there is thus likelihood for women and children to suffer from more hunger in the wake of the lockdown. Nepal’s poor scoring in the Global Hunger Index is reflective of the low minimum acceptable diet of 36% in children 6 to 23 months [7]. This is linked to a high rate of stunting and the intergenerational cycle of malnutrition. Furthermore, we can expect an increase in infants with low birth weight (LBW) due to inadequate intake of nutrients during pregnancy resulting in poor pregnant weight gain and reduced fetal nutrition. The LBW will increase morbidity and mortality among children and on survival, produce another generation of adults with lesser physical and cognitive potential. To harness the progress made so far in improving the nutritional status of women and children, it is a crucial time to prepare and put in place steps to prevent an outpouring in malnutrition, micronutrient deficiencies, and LBW babies in the near future and its repercussions on the health system and human capital. Also to mention, lockdown can be a risk factor for increased overweight in children which needs equal attention. This can be anticipated in urban areas as there is a high chance of consumption of junk foods and ultra-processed calorie-dense food leading to over nutrition and lack of physical activities during this period.

The possible ways to ensure better nutrition among women and children in resource-constrained settings like Nepal could be a combination of different measures. This includes developing and implementing mitigation strategies to reach out to those most affected by the crisis and activation and functionality of nutrition clusters at the sub-national level to ensure predictable, timely, and effective nutrition response. Similarly, program and service to protect, promote and support optimal breastfeeding and age appropriate and safe complementary feeding and feeding practices should remain a critical component of the response for women and children in the context of COVID-19. Continuity of safe motherhood services along with maternal and child nutrition interventions are required including vitamin A and deworming tablets supplementation, screening and treatment of children with acute malnutrition, distributing fortified flour to pregnant women and children above 6 months, supplying micronutrient powder, and ensuring proper counseling on infant and young child feeding practices. Similarly, mass awareness campaigns on the importance of breastfeeding, complementary feeding, and feeding during pregnancy and breastfeeding children should be done through various sources of media including mobile text messages. Counseling and psychological support to mothers and caregivers of under 5-year children is also required to promote nutrition, health, and wellbeing. Continuation of services through outpatient therapeutic centers to treat malnourished children by applying the simplified treatment approach like reducing the number of visits and by increasing the ratio of ready to use therapeutic food to be taken to the home is required to minimize the risk of contamination. Equally important is ensuring nutrition commodities are available and accessible, market supply should not be disrupted and promotion of the locally available nutritious foods should be done. In food-insecure areas where communities have limited access to adequate food, strategies such as the distribution of specialized nutritious food (e.g. supply of fortified flour) can be done.

## Conclusion

Being an issue of serious concern, it would be appropriate for Nepal and other similar resource constrained settings to focus its efforts on addressing the nutrition of women and children for building healthier societies. Focusing only on COVID-19 might bring forward other health consequences which could strain the capacity of the health system and country’s socio-economic potential. Nutrition should thus be a core component of the COVID-19 response plan, integrated into each aspect of prevention, treatment, and recovery.

## Data Availability

Not applicable.

## Abbreviations

COVID: Corona virus disease
LBW: Low birth weight
WHO: World Health Organization
